# Supplementary material for: The Cell Envelope Stress Response of Bacillus subtilis towards Laspartomycin C
Source: Antibiotics (Basel). 2020 Oct 23;9(11):729. doi: 10.3390/antibiotics9110729 (PMC7690785; doi:10.3390/antibiotics9110729)
Supplement: Supplementary file 1 [file antibiotics-09-00729-s001.pdf]

Supplementary Material to:

Article

The Cell Envelope Stress Response of *Bacillus subtilis* towards Laspartomycin C

Angelika Diehl [1,2], Thomas M. Wood [3,4], Susanne Gebhard [5], Nathaniel I. Martin [3] and Georg Fritz [2,*]

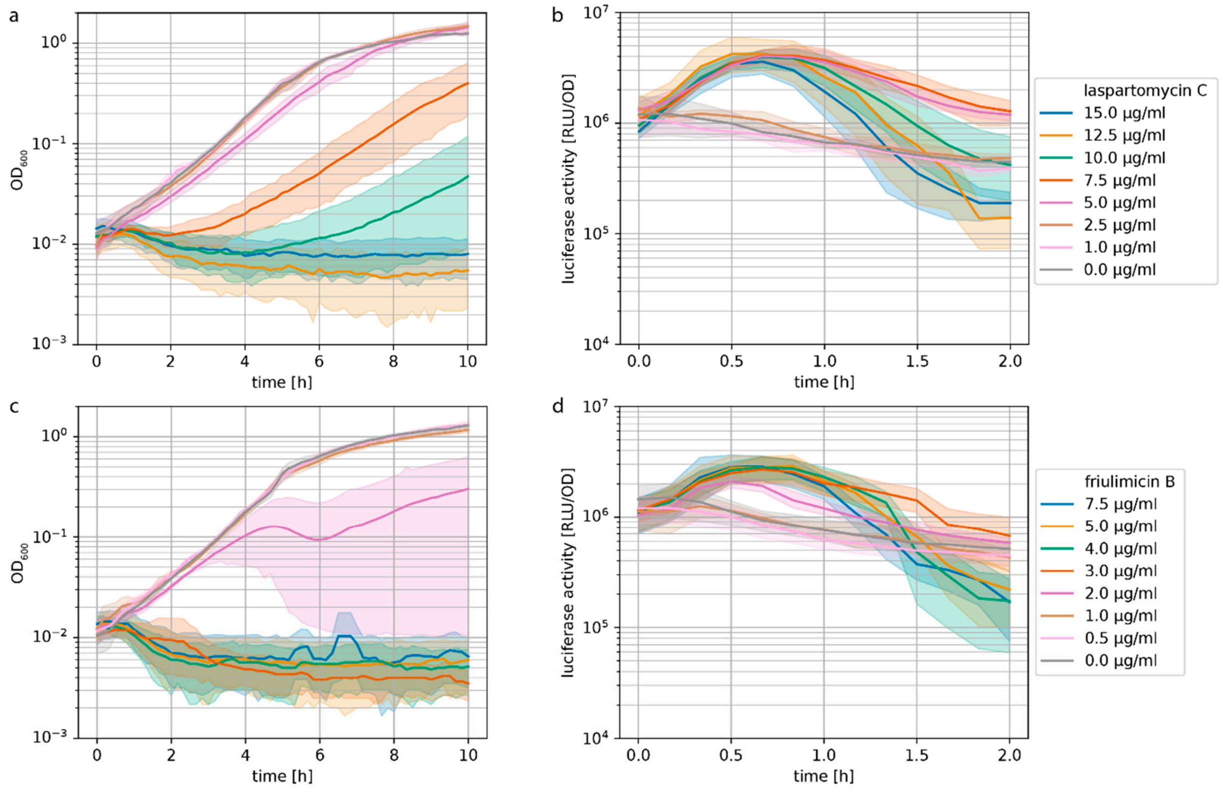

Figure S1. Kinetics of growth (a, c) and luminescence (b, d) of a *B. subtilis* strain harbouring the lux-cassette under the P_{bcrC} promoter (P_{bcrC}-luxABCDE) under laspartomycin C (a, b) and friulimicin b (c, d) challenge. Growth and luminescence are shown for the first 10 h and 2 h after antibiotic challenge, respectively. Shaded areas depict 95% confidence intervals.

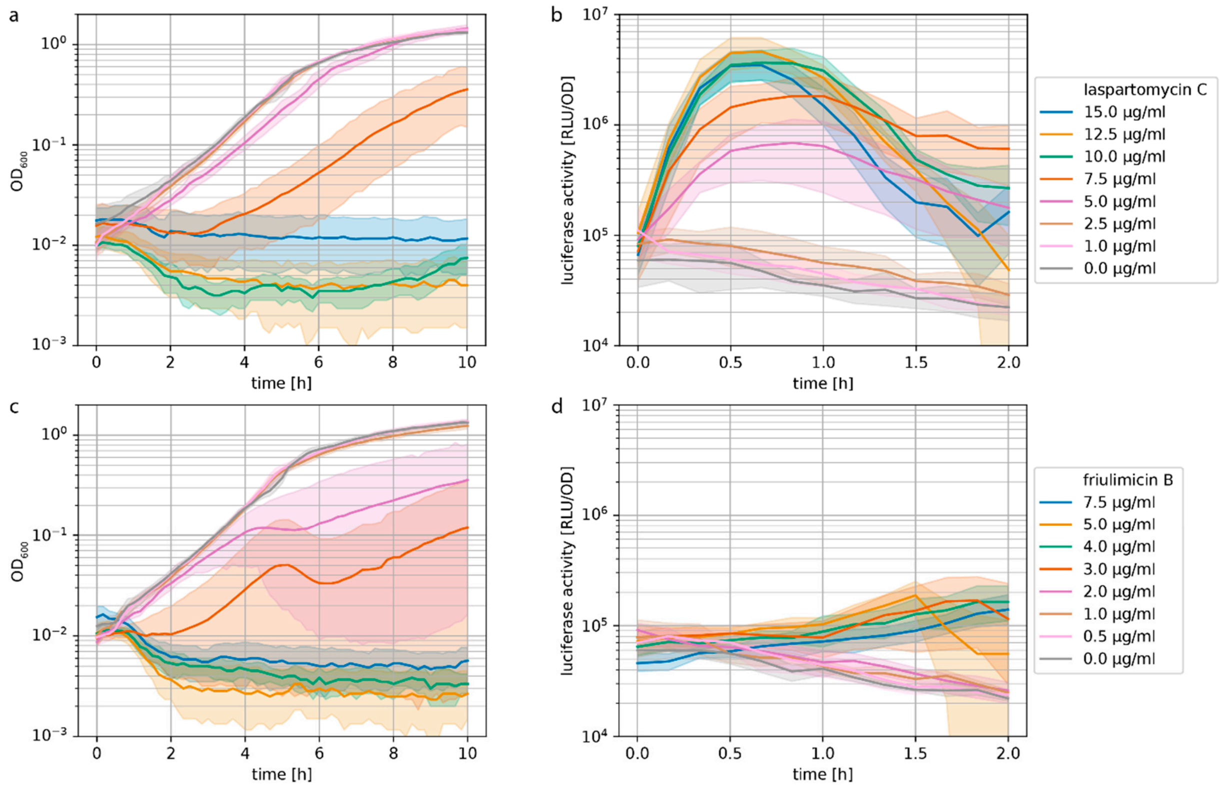

Figure S2. Kinetics of growth (a, c) and luminescence (b, d) of a *B. subtilis* strain harbouring the lux-cassette under the P_{liaI} promoter (P_{liaI}-luxABCDE) under laspartomycin C (a, b) and friulimicin b (c, d) challenge. Growth and luminescence are shown for the first 10 h and 2 h after antibiotic challenge, respectively. Shaded areas depict 95% confidence intervals.

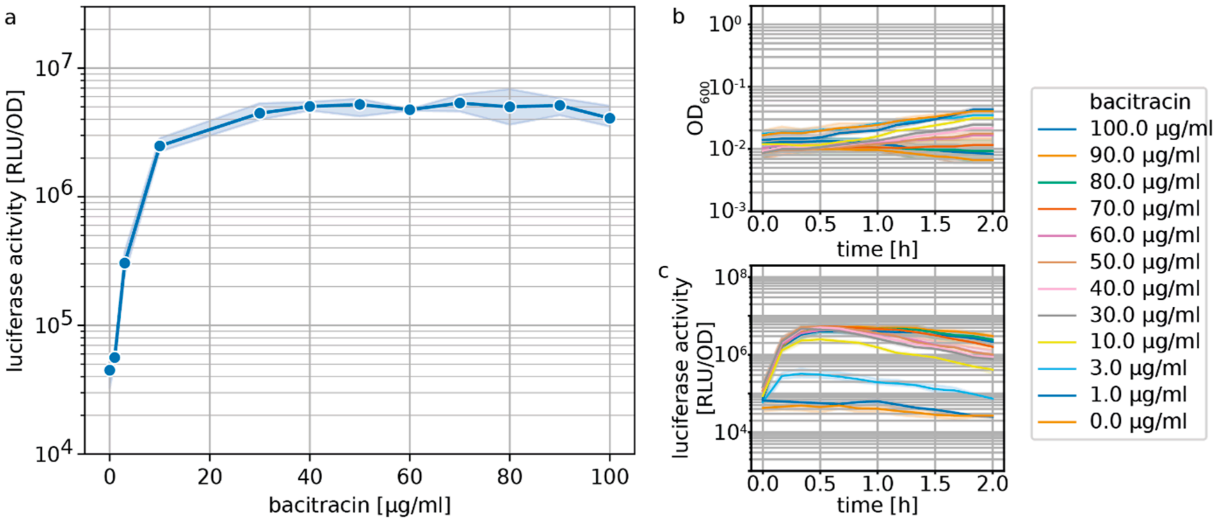

Figure S3. Response of the Lia module in response to bacitracin challenge in our setup. (a) Dose-dependent stress response of the Lia module as shown by *liaI* promoter activation during bacitracin attack. The induction at the IC_{50} is indicated in gray, and the fold-change is given. Measurements were taken 30 min after antibiotic challenge. Shaded areas depict 95% confidence intervals. Kinetics of growth (b) and luminescence (c) of a *B. subtilis* strain harbouring the lux-cassette under the P_{liaI} promoter (P_{liaI}-luxABCDE) under bacitrain challenge. Growth and luminescence are shown for the first 2 h after antibiotic challenge. Shaded areas depict 95% confidence intervals.

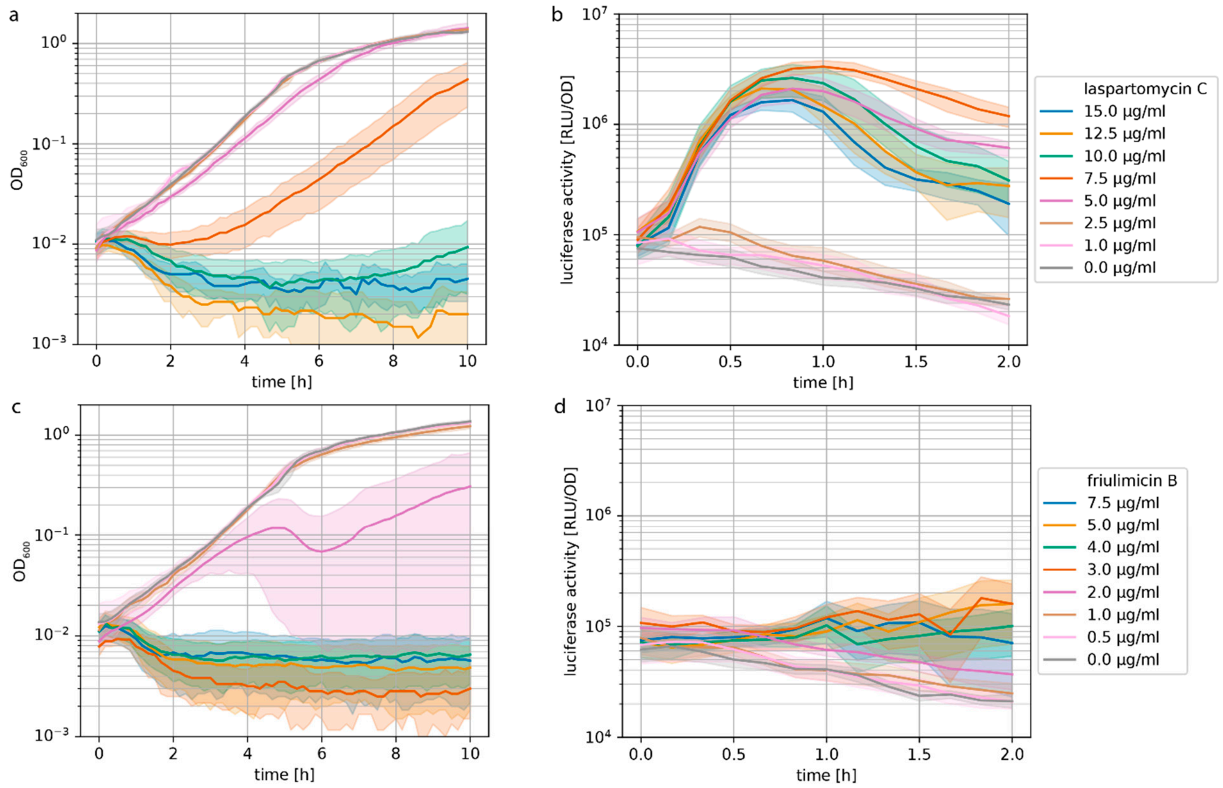

Figure S4. Kinetics of growth (a, c) and luminescence (b, d) of a *B. subtilis* strain harbouring the lux-cassette under the P_{bceA} promoter (P_{bceA}-luxABCDE) under laspartomycin C (a, b) and friulimicin b (c, d) challenge. Growth and luminescence are shown for the first 10 h and 2 h after antibiotic challenge, respectively. Shaded areas depict 95% confidence intervals.

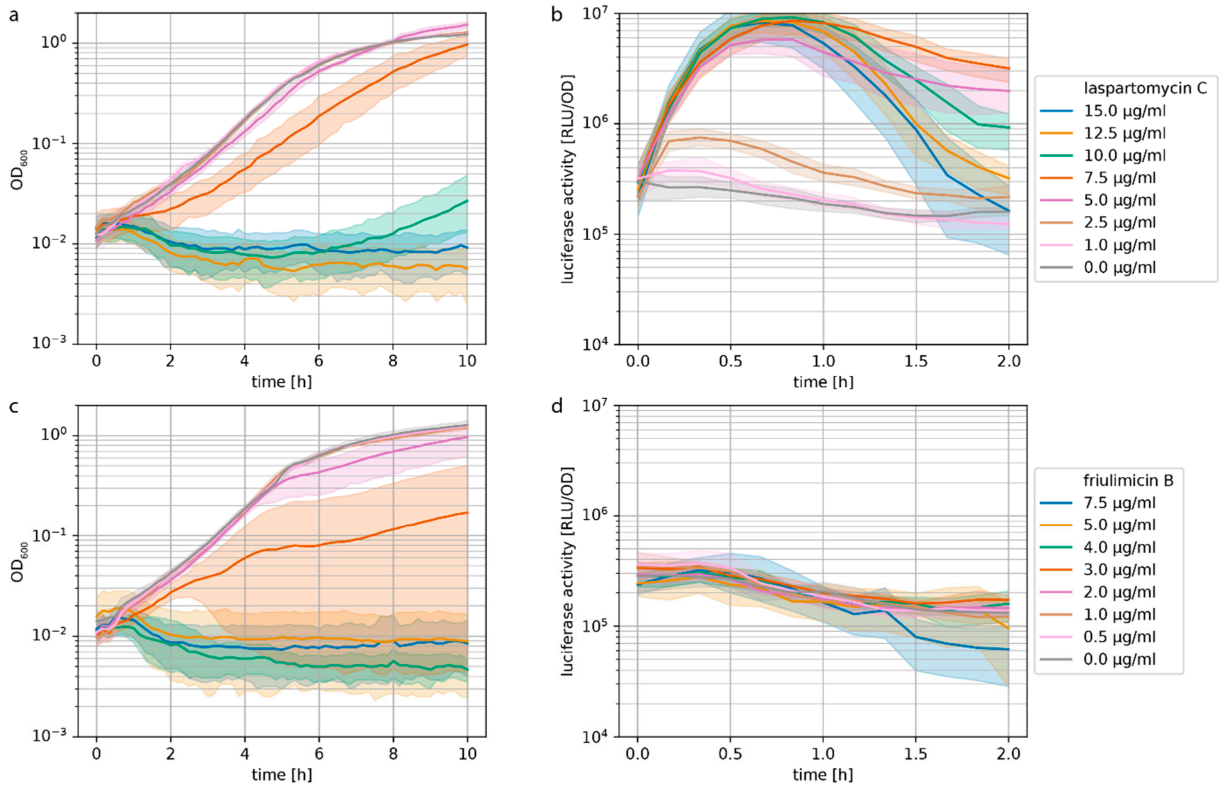

Figure S5. Kinetics of growth (a, c) and luminescence (b, d) of a *B. subtilis* strain harbouring the lux-cassette under the P_{psdA} promoter (P_{psdA}-luxABCDE) under laspartomycin C (a, b) and friulimicin b (c, d) challenge. Growth and luminescence are shown for the first 10 h and 2 h after antibiotic challenge, respectively. Shaded areas depict 95% confidence intervals.

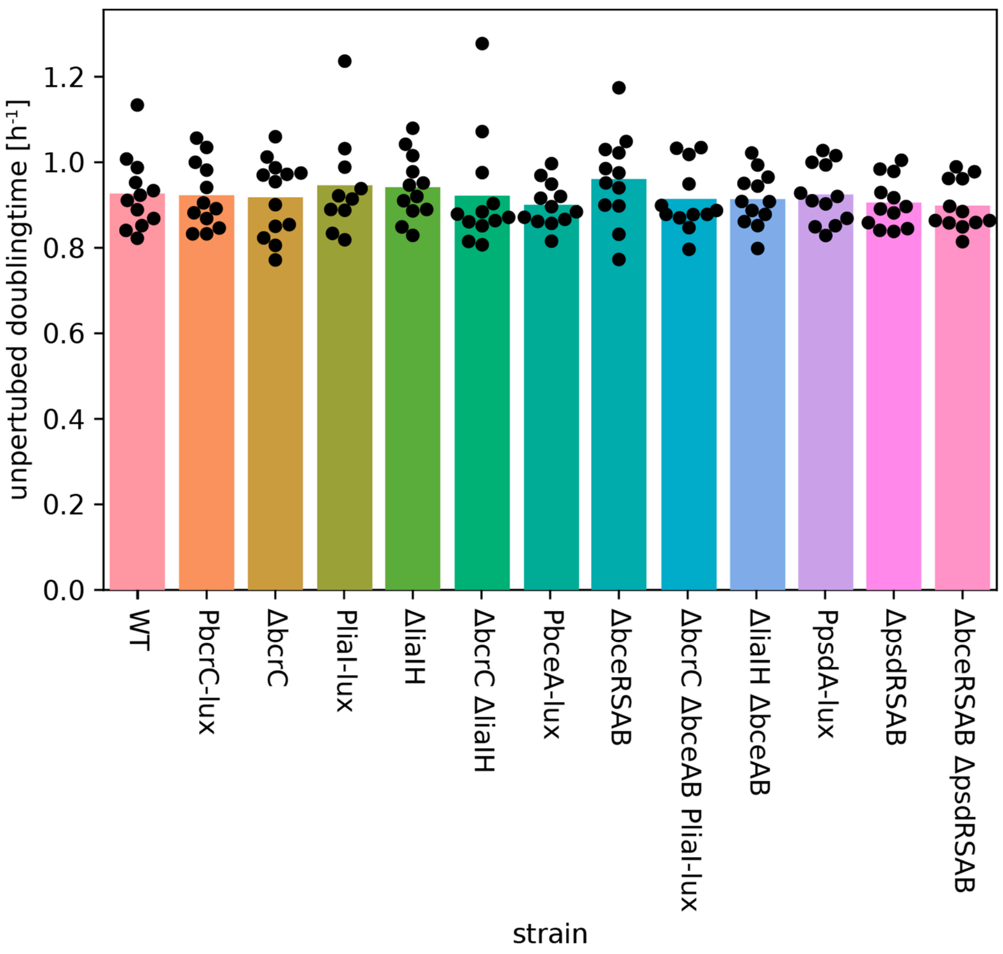

Figure S6. Unperturbed doubling time of all strains used in this study. Doubling time was determined by linear regression between the measurements reading just below OD_{600} 0.05 and just above OD_{600} 0.2. Black dots show single replicates.

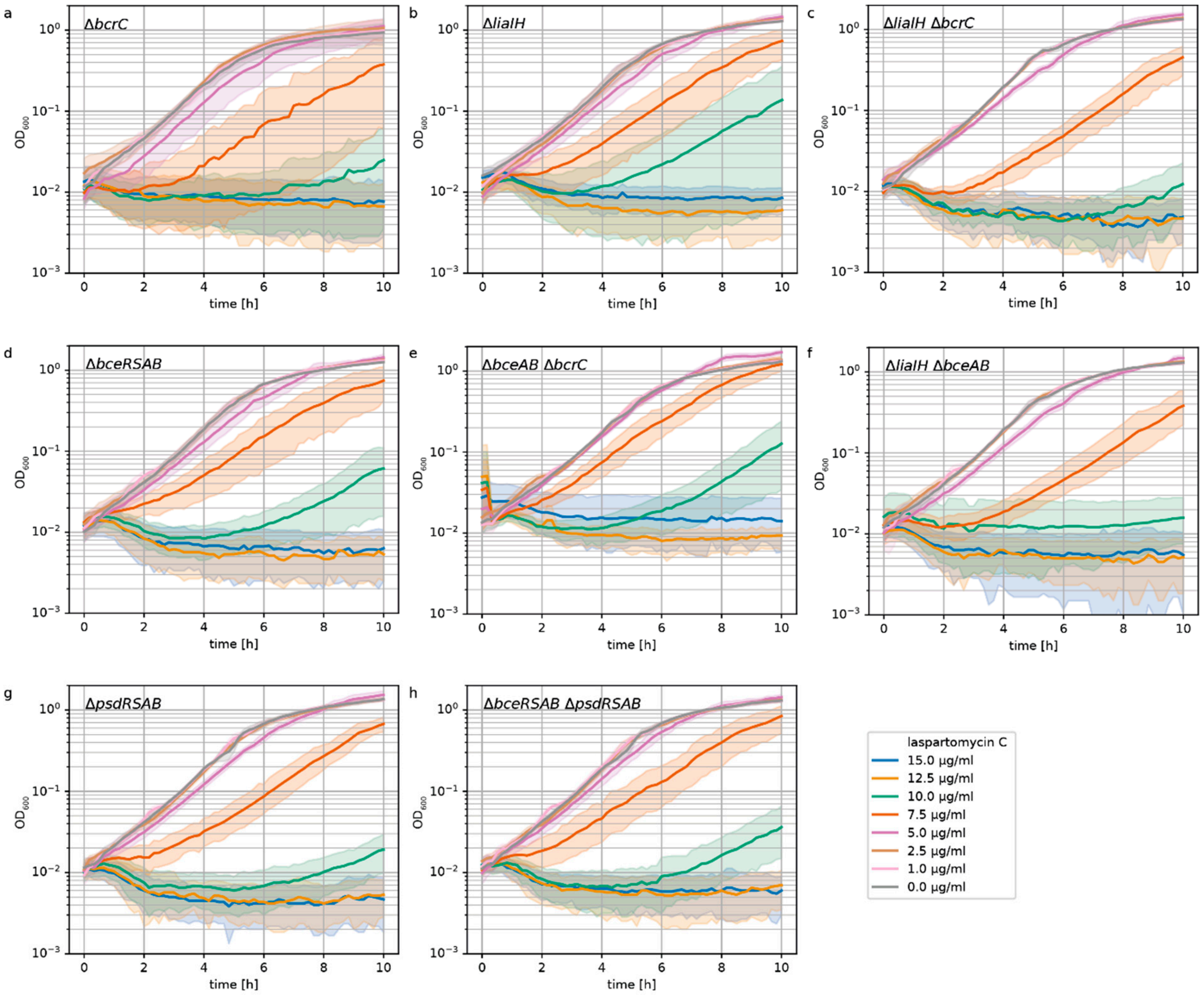

Figure S7. Growth kinetics of deletions strains challenged with laspartomycin C. The relevant genotype of strains is given in the plot. Growth is shown for the first 10 h after antibiotic challenge. Shaded areas depict 95% confidence intervals.

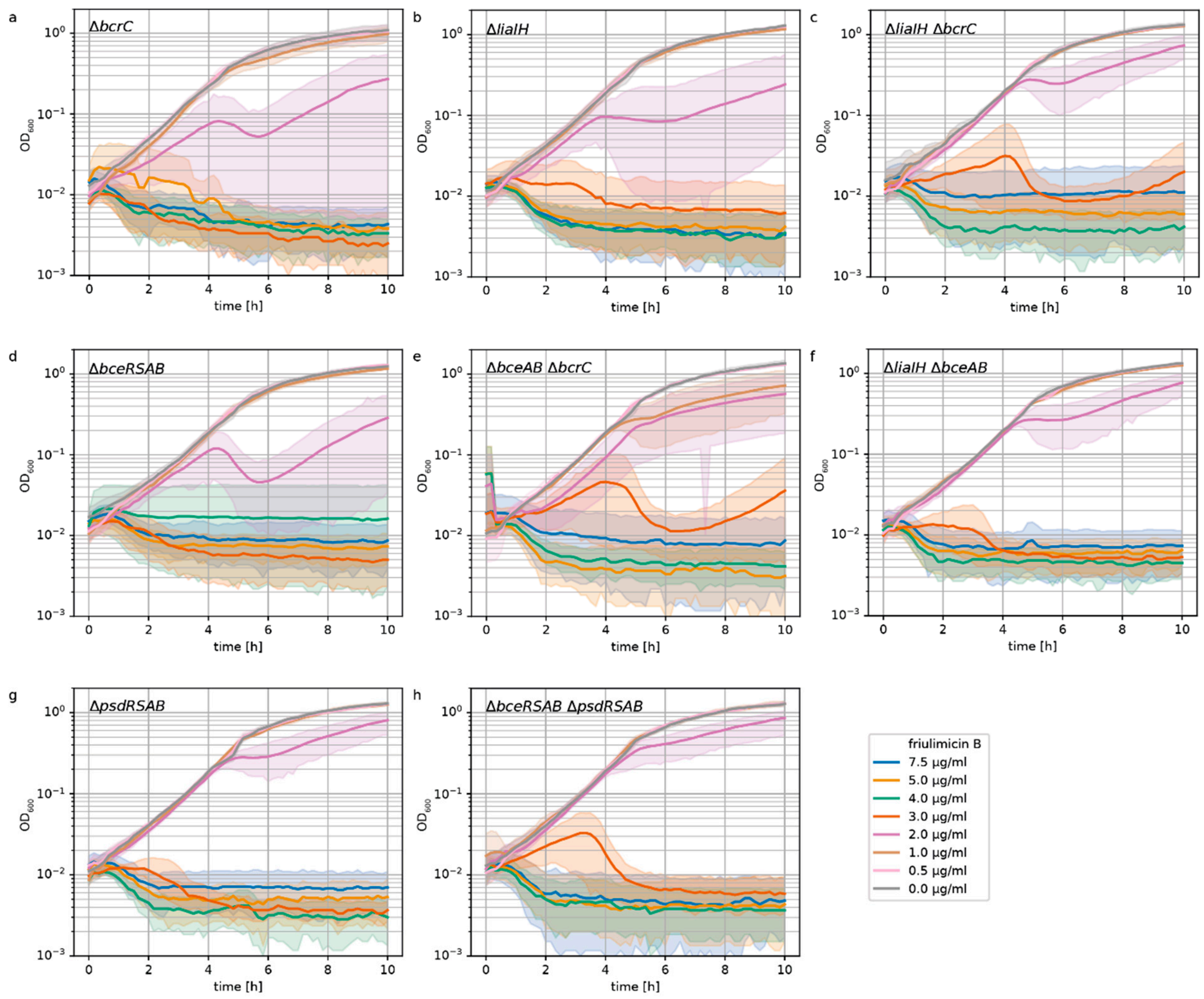

Figure S8. Growth kinetics of deletion strains challenged with friulimicin B. The relevant genotype is given in the plot. Growth is shown for the first 10 h after antibiotic challenge. Shaded areas depict 95% confidence intervals.
